# Compliance and Utility of a Smartphone App for the Detection of Exacerbations in Patients With Chronic Obstructive Pulmonary Disease: Cohort Study

**DOI:** 10.2196/15699

**Published:** 2020-03-19

**Authors:** Juan Luis Rodriguez Hermosa, Antonia Fuster Gomila, Luis Puente Maestu, Carlos Antonio Amado Diago, Francisco Javier Callejas González, Rosa Malo De Molina Ruiz, Manuel E Fuentes Ferrer, Jose Luis Álvarez Sala-Walther, Myriam Calle Rubio

**Affiliations:** 1 Pulmonology Department Hospital Clínico San Carlos Madrid Spain; 2 Medical Department, School of Medicine, Universidad Complutense de Madrid Madrid Spain; 3 Hospital Universitario Son Llátzer Palma de Mallorca Spain; 4 Pulmonology Department Hospital Universitario Gregorio Marañón Madrid Spain; 5 Pulmonology Department Hospital Universitario Marqués de Valdecilla Santander Spain; 6 Medical Department School of Medicine Universidad de Cantabria Santander Spain; 7 Pulmonology Department Complejo Hospitalario Universitario de Albacete Albacete Spain; 8 Pulmonology Department Hospital Universitario Puerta de Hierro de Majadahonda Madrid Spain; 9 Departament of Preventive Medicine Hospital Clínico San Carlos Madrid Spain; 10 Instituto de Investigación Sanitaria del Hospital Clínico San Carlos Madrid Spain

**Keywords:** chronic obstructive pulmonary disease, mHealth, compliance, mobile phone

## Abstract

**Background:**

In recent years, mobile health (mHealth)–related apps have been developed to help manage chronic diseases. Apps may allow patients with a chronic disease characterized by exacerbations, such as chronic obstructive pulmonary disease (COPD), to track and even suspect disease exacerbations, thereby facilitating self-management and prompt intervention. Nevertheless, there is insufficient evidence regarding patient compliance in the daily use of mHealth apps for chronic disease monitoring.

**Objective:**

This study aimed to provide further evidence in support of prospectively recording daily symptoms as a useful strategy to detect COPD exacerbations through the smartphone app, Prevexair. It also aimed to analyze daily compliance and the frequency and characteristics of acute exacerbations of COPD recorded using Prevexair.

**Methods:**

This is a multicenter cohort study with prospective case recruitment including 116 patients with COPD who had a documented history of frequent exacerbations and were monitored over the course of 6 months. At recruitment, the Prevexair app was installed on their smartphones, and patients were instructed on how to use the app. The information recorded in the app included symptom changes, use of medication, and use of health care resources. The patients received messages on healthy lifestyle behaviors and a record of their cumulative symptoms in the app. There was no regular contact with the research team and no mentoring process. An exacerbation was considered reported if medical attention was sought and considered unreported if it was not reported to a health care professional.

**Results:**

Overall, compliance with daily records in the app was 66.6% (120/180), with a duration compliance of 78.8%, which was similar across disease severity, age, and comorbidity variables. However, patients who were active smokers, with greater dyspnea and a diagnosis of depression and obesity had lower compliance (*P*<.05). During the study, the patients experienced a total of 262 exacerbations according to daily records in the app, 99 (37.8%) of which were reported exacerbations and 163 (62.2%) were unreported exacerbations. None of the subject-related variables were found to be significantly associated with reporting. The duration of the event and number of symptoms present during the first day were strongly associated with reporting. Despite substantial variations in the COPD Assessment Test (CAT), there was improvement only among patients with no exacerbation and those with reported exacerbations. Nevertheless, CAT scores deteriorated among patients with unreported exacerbations.

**Conclusions:**

The daily use of the Prevexair app is feasible and acceptable for patients with COPD who are motivated in their self-care because of frequent exacerbations of their disease. Monitoring through the Prevexair app showed great potential for the implementation of self-care plans and offered a better diagnosis of their chronic condition.

## Introduction

Chronic obstructive pulmonary disease (COPD) places an enormous burden on health care systems. A substantial proportion of the cost is attributable to hospitalizations, mostly owing to acute exacerbations of respiratory symptoms [1]. Acute exacerbations of COPD (AECOPD) have important consequences for patients and health care providers; AECOPD cause a negative impact on health-related quality of life [2,3], a decline in pulmonary function [4], increased utilization of health care resources [5], and decreased survival [6,7].

Knowledge about COPD exacerbation frequency is important to assess the clinical risk [8-10]. The identification and correct assessment of COPD exacerbations is vital, given that it will strongly influence therapy success and the impact on patients’ morbidity, mortality, and quality of life. Some individuals appear more susceptible to developing exacerbations and are termed frequent exacerbators or COPD exacerbator phenotypes [11,12]. Patients with frequent exacerbations are specifically targeted with more aggressive therapy and an action plan to help prevent exacerbations [8,9] and improve their quality of life [13,14]. In addition, the early identification of exacerbations by patients and early treatment may have effects on patient-reported outcomes [15]. Despite growing evidence supporting the importance of the identification and correct assessment of COPD exacerbations, less than one-third of exacerbations remain unreported to health care professionals. Unreported exacerbations are common and important events. Several studies through questionnaires showed that nearly half of all exacerbations remain unreported [16,17]. Exacerbations that are unreported and untreated by health care professionals are associated with worsening in the quality of life [3,18,19] and an increased risk of subsequent hospitalization [20,21]. Failure to seek medical attention has consequences. There is a need for new strategies to capture symptom-based exacerbations and thus provide better management of COPD.

In recent years, health-related apps running on mobile devices such as smartphones and tablets, known as mobile health (mHealth) apps, have been developed to help manage chronic diseases [22]. Current apps aid patients in managing their chronic disease, aid patients in adopting a healthy lifestyle (good nutrition, exercise, and smoking cessation), and can aid in providing a better quality of life for patients. In patients with chronic disease characterized by exacerbations, such as COPD, apps may allow tracking and even alert patients and health care professionals about suspected disease exacerbations, thereby facilitating self-management and prompt intervention [23-25]. However, maintaining continuous use is still a challenge. The use of apps to capture the reality of subjects’ lives in chronic disease is quite problematic [26,27]. The complex issues around compliance, and the relationship between symptoms and behavior, has led to the need for more research examining the parameters that contribute to daily compliance [28,29]. There is insufficient evidence regarding patient compliance in the daily use of mHealth apps for chronic disease monitoring and the determinants that contribute to this compliance. We were especially interested in these issues in the context of COPD, where little research has been done on recording symptoms daily using mHealth apps.

This study aimed to provide further evidence in support of the hypothesis that prospectively recording daily symptoms is a useful tool to monitor and help correctly assess COPD exacerbations and the clinical risk of COPD based on the patient’s daily self-reporting of symptoms using Prevexair, a simple smartphone app, in which the patient records their daily symptoms and which offers general recommendations. As there is not enough information based on previously reported results, this study was proposed as a pilot study to generate information.

This paper has provided data about compliance in the daily use of a mHealth app for the long-term monitoring of patients with COPD without a mentoring process or regular phone calls. In addition, this paper has analyzed the frequency and characteristics of AECOPD recorded via the smartphone app, Prevexair.

## Methods

### Study Patients

Patients were recruited in outpatient respiratory clinics from 6 tertiary referral hospitals in Spain between November 2016 and March 2018. The inclusion criteria were as follows: aged above 40 years, having a history of smoking (≥10 pack-years), a diagnosis of COPD confirmed by postbronchodilator spirometry with a forced expiratory volume in one second (FEV_1_) to forced vital capacity ratio of less than 0.7 in the stable phase of the disease, having a history of at least two exacerbations treated with oral corticosteroids or antibiotics or having been hospitalized at least once for exacerbation in the past 12 months, owning a smartphone, and having the cognitive and motor ability to operate a smartphone. Patients were excluded if they had other significant respiratory diseases or if they reported an exacerbation during the run-in period. Ethical approval was obtained from the Ethics Committee at the Hospital Clínico San Carlos (Madrid, Spain; internal code 14/124-E), and all patients gave their written informed consent before inclusion.

### Study Design and Patient Evaluation

This was a multicenter, prospective cohort study with a 2-week run-in period followed by a 6-month follow-up period. The study visits were scheduled as follows: before the 2-week run-in period (selection visit), after the run-in period (inclusion visit), during the follow-up period at 3 months (visit 1), and at 6 months (visit 2). The run-in period was used to make sure patients were stable. Patient assessment included a complete medical history (height, weight, smoking history, drug history, diagnosis of depression and/or anxiety, and other comorbid conditions), spirometry, health-related quality of life using the COPD Assessment Test (CAT), dyspnea using the modified Medical Research Council (mMRC) questionnaire, and the number of moderate/severe exacerbations in the last year.

### Data Collection and Monitoring

The app was developed for iOS and Android systems by using Virtual Ware. The app is available for installation on a mobile device. At recruitment, during selection visit, the Prevexair app was installed on patients’ smartphones, and they were instructed how to use the app and received instructions to record their daily respiratory symptoms in the app, once under supervision. Thereafter, in the inclusion visit, their ability to use the app was reviewed. No problems were recorded regarding the use of the mobile app.

**Figure 1:**
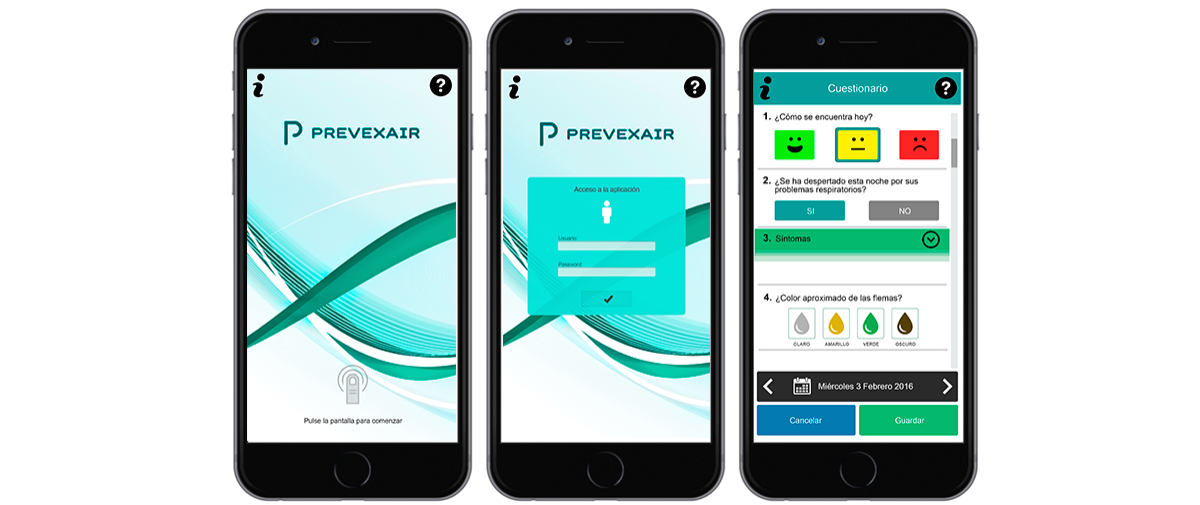
Screenshots of the Prevexair app in a smartphone: initial screens.

**Figure 2:**
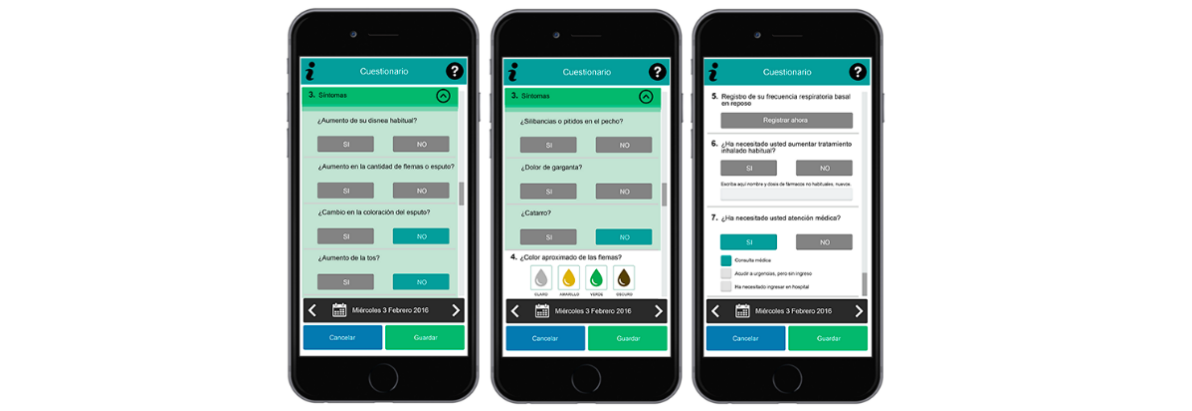
Screenshots of the Prevexair app in a smartphone: questionnaire.

The information recorded in the app included symptom changes, use of medication, and use of health care resources. Figures 1 and 2 show several screenshots of the Prevexair app in a smartphone. The following symptoms were included in the app: dyspnea, sputum color and amount, wheeze, cough, colds, and sore throat. The symptom questions had dichotomous response options, with a positive response indicating that the symptom was worse than at baseline. In addition, patients were instructed to use the app to record whether they increased their inhalation medication and started corticosteroids or antibiotics as well as any medical assistance. Daily data entries were made in the evening by setting a reminder alarm. If they forgot, patients were only allowed to enter the data for the 3 previous days. Patients automatically sent these records to a central server to be monitored in real-time by the research team. App users received messages on healthy lifestyle behaviors and disease education, information about their medication, task notifications, and a record of their cumulative symptoms in a graph through the app. Occasional contact was made, but only to solve minor technical problems. No regular contact was established, and no mentoring process was implemented to increase compliance. Participants were informed that the data sent would only be consulted by the research group; however, their physicians were blinded; they were not informed about the Prevexair app records. Thus, if they felt ill, they should contact their regular physicians for advice as usual. Decisions to change treatment or to go to the hospital will be made according to usual practice upon orders from the primary care or the respiratory specialist treating the patient, but these decisions were not based on the information provided during the study.

Additional information gathered at each scheduled visit during a clinically stable period included changes in medication, current smoking habits, CAT, and use of health care resources. During the visit at 6 months, the level of satisfaction with the app was evaluated on a scale from 0 to 10. The medical staff at the visit were blinded and not informed about the Prevexair app records.

### Data Management

To analyze data recording compliance in the app, there were 180 *available days* for each participant, including the periods of hospitalization. Two measurements of compliance were defined: overall compliance (percentage of available days that data were recorded in the app) and duration of compliance (days elapsed from the first to last app entry, as a percentage of available days).

An exacerbation was defined as an increase in respiratory symptoms for 2 consecutive days, with at least one major symptom (dyspnea, sputum purulence, or sputum volume) and either another major or minor symptom (wheeze, cold, sore throat, or cough). The first day a symptom worsened was defined as the day of onset of the exacerbation, according to the previously validated criteria [30]. Symptom counts were obtained by adding each increased respiratory symptom recorded in the Prevexair app on the first day of the exacerbation.

Exacerbation duration was defined as the number of days after onset that worsened symptoms persisted. The last day of recorded worsened symptoms before 2 consecutive symptom-free days was defined as the end of the exacerbation. Patients had to be symptom-free for ≥7 days before a new exacerbation onset was defined. Exacerbation recovery could not be determined if patients failed to record symptoms or continuously recorded symptoms for more than 30 days after onset.

An exacerbation was considered reported if medical attention was sought through scheduled or unscheduled doctor visits, emergency department visits, or hospital admissions. It was considered unreported if it was not medically reported. An exacerbation was considered treated if there was a change in at least one medication (ie, antibiotics, corticosteroids, or bronchodilators) for the worsened symptom.

To analyze the impact of exacerbation on the health status, the patients were categorized into four exclusive groups according to the reporting status for exacerbations: no exacerbation, unreported exacerbation only, reported exacerbation only, and mixed unreported (at least one) and reported (at least one) exacerbations. For this analysis, those patients with less than 60% overall compliance were excluded.

### Statistical Analysis

Qualitative variables were summarized by their frequency distribution and quantitative variables by their mean and standard deviation. Continuous nonnormally distributed variables were summarized by median and interquartile range (IQR P25-P75).

The association between the quantitative variable level of compliance and patient characteristics was evaluated with the nonparametric Mann-Whitney test for two independent groups or the Kruskal-Wallis test for more than two groups.

The association between each independent variable (baseline patient characteristics and event characteristics) and the dependent variable type of exacerbation (unreported or reported) was assessed by calculating the crude odds ratio via a multilevel logistic regression analysis. The multilevel analysis included two levels: the event level (level 1) and the patient level (level 2). A multivariable, multilevel logistic model was fitted to evaluate the independent effect of the selected variables. Candidate predictors with *P*<.10 in the univariate analysis were accepted for inclusion in the multivariate analysis.

In the study of the relationship between exacerbations and quality of life, quantitative variables were compared between the four groups in the study using the Kruskal-Wallis test and qualitative variables were compared using the chi-square test.

All analyses were performed using STATA 15.0 software (StataCorp LLC). Statistical significance was assumed at *P*<.05.

## Results

### Demographic and Clinical Information

Of the 126 patients recruited, 10 patients were excluded because they had one exacerbation at enrollment. A total of 116 patients were eligible for analysis; 21.6% (25/126) of participants were women and 13.8% (16/116) were active smokers. The baseline characteristics of the analyzed cohort are reported in Table 1. The mean (SD) FEV_1_ was 1.19 (0.47) L, and the percent predicted FEV_1_ was 44.6 (16.2). In total, 53.4% (62/116) of participants had experienced at least one severe exacerbation in the last year, and 76.7% (84/116) of participants had a degree of dyspnea ≥2 mMRC.

**Table 1:** Baseline characteristics of the study population.

Characteristics		Values
Patients, n		116
Gender (male), n (%)		91 (78.4)
Age (years), mean (SD)		66.51 (8.14)
Active smokers, n (%)		16 (13.8)
Smoking pack-years, mean (SD)		44.1 (23.60)
**BMI (kg/m^2^)**		
	Mean (SD)	27.30 (4.96)
	≤21, n (%)	9 (7.8)
**Number** **of comorbidities**		
	Mean (SD)	2.62 (1.41)
	≥3, n (%)	58 (50.0)
**Dyspnea (mMRC^a^), n (%)**		
	0-1	27 (23.2)
	≥2	89 (76.7)
CAT^b^ questionnaire, mean (SD)		14.10 (6.13)
Chronic bronchitis, n (%)		61 (52.6)
Chronic colonization, n (%)		2 (1.7)
History of asthma, n (%)		7 (6.0)
Post-BD FEV_1_ (%)^c^, mean (SD)		44.62 (16.23)
Post-BD FEV_1_ (mL), mean (SD)		1192.11 (477.16)
Number of severe-moderate exacerbations^d^ in the last year, median (IQR P25-75)		3(2-3)
Number of moderate exacerbations in the last year ≥2, n (%)		65 (56.0)
Number of severe exacerbations in the last year, median (IQR P25-75)		1 (0-2)
Number of severe exacerbations in the last year ≥1, n (%)		62 (53.4)
**Drug treatment for COPD^e ^, n (%)**		
	LAMA^f^ monotherapy	6 (5.2)
	LAMA-LABA^g^ combination	37 (31.9)
	LABA+ICS^h^ combination	6 (5.2)
	Triple therapy^i^	67 (57.7)
Long-term oxygen therapy, n (%)		36 (31.0)
Chronic antibiotics, n (%)		1 (0.9)

^a^mMRC: modified Medical Research Council.

^b^CAT: Chronic Obstructive Pulmonary Disease Assessment Test.

^c^Post-BD FEV_1_ %: postbronchodilator FEV_1_ percent predicted.

^d^Severe exacerbations refers to exacerbations requiring hospitalization; moderate exacerbations refer to exacerbations requiring outpatient management with antibiotics and/or corticosteroids systemic.

^e^COPD: chronic obstructive pulmonary disease.

^f^LAMA: long-acting antimuscarinic agent.

^g^LABA: long-acting beta-2 agonist.

^h^ICS: inhaled corticosteroid.

^i^Triple therapy: LABA+LAMA+ICS.

### Daily Compliance

The 116 patients recorded data in the Prevexair app for a median of 178 (IQR 130-180) days, while the median number of records per patient was 120 (IQR 61-164).

Overall compliance in recording data daily in the app was 66.6% (120/180), with a compliance duration of 78.8%. Compliance is reported in Table 2. Overall and compliance duration rates were similar across the disease severity, age, and comorbidity variables. However, patients who are active smokers, with a higher mMRC functional dyspnea score and a diagnosis of depression and obesity, had lower overall compliance and duration of compliance (*P<*.05). Female participants also had a lower duration of compliance. Furthermore, participants with a higher degree of satisfaction with the app had better overall compliance and duration of compliance (*P<*.05).

The percentage of patients who discontinued their use of the app was 6% during the first month and 8.6% during the second and third months, and 12.9% of patients abandoned the app during the last 3 months. The median (P25-75) level of satisfaction with the app was 10 (8-10).

**Table 2:** Daily compliance by clinical variable.

Characteristics		Participants, n	Overall compliance^a^, median (IQR)	*P* value	Compliance duration^c^, median (IQR)	*P* value
All subjects		116	66.6 (33.8-91.1)	N/A^b^	78.8 (51.5-94.9)	N/A
**Sex**				.10		.03
	Male	91	71.6 (35.5-93.3)		82.7 (56.7-95.6)	
	Female	25	53.3 (31.9-79.1)		74.4 (42.1-85.2)	
**Age (years)**				.79		.88
	<65	44	63.8 (34.8-90.5)		78.1 (45.1-95.5)	
	≥65	72	67.5 (33.8-91.9)		79.4 (56.7-92.7)	
**BMI (kg/m^2 ^)**				.02		.01
	25-26.9	40	78.6 (42.2-95.4)		80 (59.8-95.4)	
	27-29.9	50	72.2 (42.9-92.2)		85.7 (64.7-96.3)	
	≥30	26	40.5 (11.2-75.5)		54.5 (32.0-85.6)	
**Smoking status**				.03		.02
	Active smoker	16	40.5 (7.9 -72.6)		54 (30.4-84.6)	
	Former smoker	100	71.6 (40.0-91.9)		81.6 (56.7-95.0)	
**Number of comorbidities**				.88		.37
	<3	58	64.1 (40.6-89.7)		75.3 (53.8-93.1)	
	≥3	58	72.2 (31.1-92.3)		84.2 (45.3-95.8)	
**Chronic bronchitis**				.57		.21
	Presence	61	64.4 (32.2-89.4)		77.9 (44.9-92.0)	
	Absence	55	68.3 (39.4-95.0)		85.5 (60.5-96.2)	
**History of asthma**				.86		.66
	Presence	12	59.1 (35.9-89.8)		70.2 (55.0-90.1)	
	Absence	104	67.5 (33.8-91.1)		80 (50.9-94.9)	
**Chronic colonization**				.57		.21
	Presence	61	64.4 (32.2-89.4)		77.9 (44.9-92.0)	
	Absence	55	68.3 (39.4-95.0)		85.5 (60.5-96.2)	
**Dyspnea (mMRC^d^)**				.08		.04
	0-1	27	80.5 (45-98.8)		87.9 (61.3-100)	
	≥2	89	63.8 (32.2-90.5)		75.6 (47.1-91.9)	
**CAT^e^questionnaire**				.76		.97
	<10	31	61.6 (41.6-92.7)		76.5 (50-96.7)	
	≥10	84	70 (33.3-90.5)		80.5 (54.5-92.6)	
**Anxiety**				.13		.34
	Presence	21	88.3(47.5-93.6)		88.3 (52.3-94.3)	
	Absence	95	63.8 (33.3-90.5)		75.7 (51.3-94.9)	
**Depression**				.008		.01
	Presence	7	12.2 (3.3-53.3)		32.1 (9.2 -87.2)	
	Absence	109	71.6 (39.7-91.6)		80.5 (56.6-95.0)	
**Post-BD FEV_1 _(%)^f^**				.78		.65
	≥50	37	71.6 (36.6-88.8)		81.6 (46.5-91.8)	
	<50	79	64.4 (33.3-92.2)		77.9 (53.8-95.1)	
**Number** **of moderate/severe exacerbations^g ^** **in the last year**				.30		.74
	<2	11	51.6 (7.2- 86.6)		80.6 (61.1-94.2)	
	≥2	105	68.3 (37.5-91.1)		78.8 (50.6-94.9)	
**Drug treatment for COPD^h ^**				.42		.64
	No triple therapy	49	71.6 (47.2-90.0)		78.8 (55.0-90.9)	
	Triple therapy^i^	67	66.6 (25.5-92.2)		80.2 (45.0-95.1)	
**Long-term oxygen therapy**				.21		.10
	Treated	36	80.5 (33.1-95.0)		87.1 (71.5-95.0)	
	Not treated	80	63.3 (33.8-90.2)		73.6 (45.8-92.3)	
**Satisfaction score**				.10		.20
	<10	50	63.8 (32.5-90.5)		73.6 (47.5-90.8)	
	≥10	54	78.6 (44.0-93.7)		85.6 (55.4-96.2)	

^a^Compliance is expressed as median percentage (number of days completed/total number of days available for completion). Overall compliance: percentage of days in the entire study period (180 days) in which the app was used daily.

^b^Not applicable.

^c^Compliance duration: days elapsed from first daily entry to last, as the percentage of days available.

^d^mMRC: modified Medical Research Council.

^e^CAT: Chronic Obstructive Pulmonary Disease Assessment Test.

^f^Post-BD FEV_1_ %: postbronchodilator FEV_1_ percent predicted.

^g^Severe exacerbations refer to exacerbations requiring hospitalization; moderate exacerbations refer to exacerbations requiring outpatient management with antibiotics and/or corticosteroids systemic.

^h^COPD: chronic obstructive pulmonary disease.

^i^Triple therapy: long-acting beta-2 agonists + long-acting antimuscarinic agents + inhaled corticosteroids.

### Exacerbations

During the study, patients experienced a total of 262 cases of symptom worsening, meeting the definition of exacerbation according to daily records in the app. The overall estimated rate of exacerbations recorded in the app was 2.25 (1.66) per person every 6 months. Of 116 patients, 18 (15.5%) had no events, 26 (22.4%) had one event, 25 (21.6%) patients had 2 events, and 47 (41.6%) patients had more than 2 events during the 6 months. Of 262 cases, 99 (37.8%) were reported exacerbations and 163 (62.2%) were unreported exacerbations.

Table 3 presents the characteristics of reported and unreported exacerbations and their relationship with the probability of reporting. In general, reported exacerbations were longer and had more symptoms: among those exacerbations with 2 symptoms present at onset, only 15.2% were reported, whereas 46.5% of those with 4 or more symptoms were reported. In reported exacerbations, sputum color (54.5% vs 22.7%; *P*<.001) and cough (74% vs 60.1%; *P*<.02) were also more common. Reporting was related to the duration and number of worsened symptoms as well as the type of symptoms present when symptoms worsened.

Of the 163 unreported exacerbations, 76 (46.6%) were treated, but all were self-managed by the patient with an increase in bronchodilators in 57 events (35.0%), only antibiotics in 10 events (6.1%), and only oral corticosteroids in 9 (5.5%) events. Of the 99 reported exacerbations, all were treated: 15 (15%) events only with an increase in bronchodilators and the majority with only antibiotics (47/99, 47.5%) or with both oral corticosteroids and antibiotics (28/99, 28.3%). With regard to recorded health care utilization for exacerbation, 79.7% of exacerbations led to unscheduled contact, 2.2% led to emergency department visits, and 18.1% of exacerbations resulted in hospitalization.

**Table 3:** Characteristics of unreported and reported exacerbations and the relationship between event characteristics and the likelihood of reporting an exacerbation.

Characteristics of exacerbations		Global	Unreported	Reported	Odds ratio (95% CI)	*P* value
Exacerbations, n (%)		262 (100.0)	163 (62.2)	99 (37.8)	N/A^a^	N/A
Duration of worsened symptoms, day median (P25-75)		6 (4-9)	5 (3-8)	8 (6-11.2)	1.17 (1.08-1.27)	<.001
Total number of key symptoms, mean (SD)		3.12 (1.09)	2.91 (1.04)	3.47 (1.09)	1.88 (1.35-2.63)	<.001
**Type of symptoms, n (%)**						
	Dyspnea	137 (52.3)	87 (53.4)	50 (50.5)	0.95 (0.49-1.00)	.9
	Sputum amount	177 (67.6)	106 (65.0)	71 (71.7)	1.48 (0.72-3.05)	.28
	Sputum color	91 (34.7)	37 (22.7)	54 (54.5)	8.39 (3.31-21.22)	<.001
	Cough	172 (65.6)	98 (60.1)	74 (74.7)	2.30 (1.13-4.66)	.02
	Wheeze	75 (28.6)	45 (27.6)	30 (30.3)	1.31 (0.62-2.75)	.47
	Sore throat	57 (21.8)	37 (22.7)	20 (20.2)	0.69 (0.29-1.63)	.49
	Cold	110 (41.9)	65 (39.9)	45 (45.5)	1.30 (0.64-2.61)	.46
**Severity by number of symptoms, n (%)**					2.93 (1.25-6.85)	<.001
	2	85 (32.5)	68 (41.7)	17 (17.1)		
	3	96 (36.6)	60 (36.8)	36 (36.4)		
	4 or more	81 (30.9)	35 (21.5)	46 (46.5)		

^a^Not applicable.

### Baseline Characteristics of Patients by Exacerbation Category

Table 4 shows that patients with reported exacerbations present similar baseline characteristics compared with patients with unreported exacerbations, although patients with reported exacerbations had more dyspnea, severe disease FEV_1_%, and anxiety. Reporting did not appear to be related to patient characteristics.

**Table 4:** Baseline characteristics of patients by exacerbation category.

Characteristics		Unreported exacerbations	Reported exacerbations	Odds ratio (95% CI)	*P* value
Gender (male), n (%)		123 (75.4)	82 (83)	1.61 (0.61-4.29)	.33
**Age (years)**					
	Mean (SD)	65.5 (8.7)	67.6 (8.3)	1.04 (0.58-4.68)	.08
	≥65, n (%)	102 (62.5)	68 (69)	1.60 (0.67-3.81)	.28
Active smokers, n (%)		27 (16.6)	12 (12)	1.68 (0.53-5.26)	.37
BMI (kg/m^2^), mean (SD)		26.6 (5.0)	26.8 (4.6)	1.02 (0.94-1.11)	.55
Number of comorbidities; mean (SD) ≥3, n (%)		68 (41.7)	55 (56)	2.11 (0.94-4.73)	.07
Depression, n (%)		8 (4.9)	3 (3)	0.55 (0.07-3.97)	.56
Anxiety, n (%)		25 (15.3)	26 (26)	2.77 (0.99-7.70)	.05
Dyspnea (mMRC^a^) ≥2, n (%)		129 (79.1)	87 (88)	2.73 (0.88-8.41)	.08
CAT^b^ questionnaire score ≥10, n (%)		115 (70.5)	80 (81)	2.28 (0.88-5.94)	.09
Post-BD FEV_1_ %^c^ predicted <50%, n (%)		114 (69.9)	74 (75)	1.58 (0.64-3.91)	.32
Number of moderate-severe exacerbations in the last year ≥2, n (%)		149 (91.4)	96 (97)	4.06 (0.69-23.72)	.12

^a^mMRC: modified Medical Research Council.

^b^CAT: Chronic Obstructive Pulmonary Disease Assessment Test.

^c^Post-BD FEV_1_ %: postbronchodilator FEV_1_ percent predicted.

### Predictors of Reporting an Exacerbation

Table 5 shows the relationship between exacerbation characteristics and the subject and the likelihood of reporting an exacerbation. None of the subject-related variables were found to be significantly associated with reporting. The duration of the event and number of symptoms present during the first day were strongly associated with reporting.

**Table 5:** The relationship between exacerbation characteristics and the subject and the likelihood of reporting an exacerbation.

Characteristics		Odds ratio (95% CI)	*P* value
Duration of worsened symptoms		1.15 (1.06-1.25)	<.001
Mean number of key symptoms		1.75 (1.20-2.56)	.003
**Number** **of comorbidities**			.45
	≥3	1.50 (0.51-4.41)	
	<3	1	
**CAT^a ^questionnaire score**			.52
	≥10	1.49 (0.44-5.00)	
	<10	1	
**Dyspnea (mMRCM^b^)**			.42
	≥2	1.75 (0.44-6.99)	
	<2	1	
**Anxiety**			.20
	Present	2.43 (0.62-9.50)	
	Not present	1	

^a^CAT: Chronic Obstructive Pulmonary Disease Assessment Test.

^b^mMRC: modified Medical Research Council.

### Impact of Exacerbations on Health Status

On average, CAT scores were worse at the end of the study period (6 months). The median (P25-75) change in CAT score was 1 (−3-4). Table 6 shows the distribution of CAT score changes between the inclusion and 6-month visits stratified by the presence and type of exacerbation recorded in the app among 69 patients who had more than 60% overall compliance. Of the 69 patients analyzed, only 6 (9%) did not have exacerbations, 19 (27%) had only unreported exacerbations, 18 (26%) had only reported exacerbations, and 26 (38%) had mixed exacerbations. Despite substantial variation in the CAT score, there was improvement among patients with no exacerbations and those with only reported exacerbations. CAT scores deteriorated in patients with unreported exacerbations. This deterioration was highest in patients who had at least one unreported exacerbation and at least one reported exacerbation. There was a difference between those with only unreported exacerbations and those with only reported exacerbations (*P*<.05). Patients in the mixed exacerbation group had statistically worse deterioration (*P*<.01) of the CAT score than those with only reported exacerbations. Deterioration of the CAT score was clinically significant (an increase of 2 or more) in 44% (8/18) of patients who did not report any of their exacerbations compared with 23% (4/18) of those with only reported exacerbations and 73% (19/26) of those with mixed exacerbations.

**Table 6:** Change in health status between inclusion and 6-month visits according to the presence and type of exacerbation during the study as recorded in the app.

Change in health status	Stable disease^a^	Unreported^b^	Reported^c^	Mixed^d^	*P* value
Subjects, n (%)	6 (9)	19 (28)	18 (26)	26 (38)	N/A^e^
Change in CAT^f^ score, median (P25-75)	−3 (−3.5-3)	1 (−2.2-6.2)	−2 (−7-1.5)	3 (0-5.2)	<.001
Patients with change in CAT score ≥2, n (%)	1 (20)	8 (44)	4 (23)	19 (73)	<.001

^a^No exacerbation between inclusion and 6-month visits.

^b^Only unreported exacerbation(s) between inclusion and 6-month visits.

^c^Only reported exacerbation(s) between inclusion and 6-month visits.

^d^At least one unreported exacerbation and one reported exacerbation between inclusion and 6-month visits.

^e^Not applicable.

^f^CAT: Chronic Obstructive Pulmonary Disease Assessment Test.

## Discussion

### Principal Findings

This study provides information about the long-term, consistent use of an mHealth app, Prevexair, to record daily symptoms and detect exacerbations in high-risk patients with COPD, as well as to determine the characteristics of the detected exacerbations and the determinants of reporting them.

The mHealth app market is booming and will continue to grow substantially over the next few years. The growing availability of health apps and the increasing number of patients using smartphones and tablets will encourage health care professionals to incorporate apps into their management plans for patients with chronic disease. This is a step toward ubiquitous health care, thereby allowing patients with chronic disease to self-manage their condition by providing them support to monitor and interpret their own data using mobile devices.

COPD is a highly prevalent disease, occurring in 10% of the population between the ages of 40 and 80 years [31]. It is a progressive disease that is frequently associated with a high rate of morbidity and mortality and is currently the fifth leading cause of death in Spain [32,33]. It is currently included in the priority plans for health care systems [34] owing to its association with a significant demand for care because of its high complexity and frequent decompensations [35]. COPD is one of the main reasons for medical consultations and the use of health care resources, both in primary and specialized care. In Spain, the disease accounts for 10% to 12% of all primary care visits, 35% to 40% of pulmonology consults, and 7% of hospitalizations [36,37]. These characteristics of COPD force us to make a change in the care model, focusing on monitoring the disease and giving the patient a part of the responsibility in managing their disease through the use of information and communication technology as a tool that has been proven useful in the self-care and monitoring of patients with COPD to detect decompensations of the disease.

### Previous Studies

Research has shown that effective management of COPD through integrated care systems, mHealth apps, and other technology has the potential to both benefit the patient and reduce exacerbation costs in the long-term management of the disease [38,39]. Several studies on action plans focusing on the early identification of exacerbations by patients and the implementation of an action plan have shown effects on health care utilization as well as on patient-reported outcomes [40-42]. The telemonitoring of a patient’s condition, symptoms, and behavior (adherence to medication and physical activity) through mHealth apps may be useful in identifying and correctly assessing COPD exacerbations, reducing the number of unreported exacerbations, and allowing the implementation of self-management. Providing the patient with the right care at the right time is crucial and can have a decisive impact on handling the long-term condition to prevent exacerbations and improve the quality of life in patients with COPD. As a result, mHealth apps are extensively used in health services and patient education. Indeed, the UK Department of Health has recommended that apps be *prescribed* as part of the care for long-term conditions [43]. However, there are few published studies addressing daily compliance in mHealth apps and what factors influence compliance.

The results of our study show a high rate of daily use of the app, Prevexair, although there was no contact between the research team and the patient after initiation and no strategy was implemented to continue using the app.

In COPD, little research has been done on diary-keeping, even though diaries have been widely used in studies and clinical trials. In an open, observational study, only 41% of participants achieved 80% compliance using paper diaries that were collected weekly and entered electronically [44]. The compliance was higher (53% in 12 months) in another study owing to a mentoring process with regular phone calls [45].

### Interpretation of Novel Findings

In our study, the level of satisfaction with the functionality of the Prevexair app was high. The patients quickly learned how to use the app during the inclusion visit and regularly entered data to record their symptoms and medicine use over 6 months, although they did not make decisions based on information provided by the app during the study. Decisions to change treatment or go to the hospital were made according to usual practice upon orders from the primary care or respiratory specialist treating the patient. Studies that have evaluated feedback from users regarding the functionality and usability of a mobile phone app show us that simplicity and motivation, not age, seem to be the key factors for accepting and using health apps [46]. With regard to these determinants of use, it is worth mentioning that the patients who participated in our study were motivated in their self-care because of frequent decompensations of their disease, with hospitalization for COPD occurring in more than half of the patients evaluated. There is evidence that participants will tolerate the burden of diary-keeping if they feel it will help them [47,48]. In this regard, it should be mentioned that the Prevexair app sent messages about healthy lifestyle behaviors and information about the patient’s medication, task notifications, and a record of their cumulative symptoms in a graph through the app, which has been found to have a compliance advantage. Studies show that users value being in charge of their health and keeping track of their progress [49]. However, self-motivation to record data over a longer period can be a challenge without the involvement of a health care professional [50,51]. There is also evidence that participants in research studies will take on additional burdens for altruistic reasons unrelated to their chronic illness [52].

### Factors Associated With Compliance

With regard to the factors related to continued daily use of the app, in our study, we did not find any differences according to age or sex, comorbidity burden, or disease severity. These results are consistent with other studies that showed that compliance rates were similar across the demographic variables of sex and disease severity [45,46]. Several studies have shown that there is no general correlation between diary-keeping and symptom severity, demographic or clinical characteristics, treatment, or activity [45,53].

We have provided new data on use and adherence to a mobile phone app for COPD. The compliance is not affected by demographic factors or disease severity, while clinical or physiological characteristics, such as actively smoked, higher BMI, or were diagnosed with depression, do seem to influence diary use. Nevertheless, a limitation to bear in mind is that other factors related to adherence such as health literacy, prior use of apps, and level of school education could not be evaluated as they were not available.

Simplicity and motivation seem to be the key factors for accepting and using mobile phone apps. However, each user has different needs, so it is important to be able to personalize the app to the patient’s preferences. So, in the patients where we identify factors linked to lower adherence, it is important to offer specific messages such as exercise tracking, monitoring of weight, food intake, and help for tobacco cessation. In addition, personalized self-management plans could be updated according to patients’ needs. Other functionality of interest can be email messaging or any type of communication with health care providers.

### Detection of Exacerbations

Regarding the detection of exacerbations by recording symptoms in the app, it is necessary to highlight the high rate of daily exacerbations and that 62% of the events recorded in the app were not reported and most were not treated. This result is similar to results of earlier studies in London [16,19] and Canada [17]. These results support the management of COPD through mHealth apps and other technology, considering that COPD is a highly symptomatic disease, but patients may not recognize small day-to-day variations in their pulmonary symptoms. Lack of symptom awareness and the rate of symptom worsening make the daily monitoring of patients with COPD an attractive and beneficial approach to detect patients with frequent exacerbations and to carry out more aggressive therapy and implement preventive measures. In addition, these technologies would allow health care professionals to monitor patients and offer opportunities for an intervention to improve outcomes. They could view patients’ data consistently, and not only periodically at the outpatient clinics. Furthermore, they could provide the patient with information to implement self-care and early treatment plans for COPD decompensations.

In our study, although unreported exacerbations tend to be milder (with a lower number of symptoms and shorter duration of exacerbation), these unreported exacerbations have a clinically relevant negative impact on quality of life (CAT questionnaire) and result in a change in self-administered treatment by the patient in a large number of cases. Patients who did not report their exacerbation were more likely to experience worsening of their health status compared with those who reported exacerbations or those with a stable disease. This may suggest that unreported exacerbations may thus represent an unmet health care need. These results are consistent with other studies, which have shown that unreported exacerbations, despite being associated with less symptom worsening than reported exacerbations, have an important medium to long-term impact on patients’ quality of life [15,18,19]. Failure to seek medical attention may have consequences for both the patient and health care system.

In our study, the characteristics of the exacerbations were the strongest predictors of reporting. Although there was no direct measure of exacerbation severity, the total number of symptoms at onset and duration of the exacerbation were predictors of reporting. The symptoms associated with reporting an exacerbation in this study were cough and change in sputum color. Sputum color was identified as one of the key determinants of health care utilization in a study looking at patient perspectives on exacerbations [54]. Although in our study no subject-related variables were found to be significantly associated with reporting, patients who reported their exacerbation had more dyspnea, anxiety, and more spirometric obstruction compared with those who did not report exacerbations. Other studies have also found that patients with a lower FEV_1_ were more likely to seek medical attention [17,55]. This is also consistent with the observation showing that physician-rated exacerbation severity correlates with the severity of the underlying disease [56]. The finding that patients with anxiety were more likely to seek medical attention may be explained by the fact that it plays a role in symptom development and patient behavior and should be considered a potentially important predictor. In other studies, psychological factors have been found to be related to the reporting of respiratory symptoms [54,57].

The identification and correct assessment of COPD exacerbations is important to assess clinical risk and disease control, a goal that is key especially in patients who appear more susceptible to developing exacerbations and are termed frequent exacerbators, similar to our study population, in which monitoring through the app, Prevexair, can be more beneficial.

The app was developed for better lifestyle management for patients with COPD and also to improve monitoring and follow-up by their physicians. In the future, we would like to analyze the usefulness of the app, Prevexair, for physicians during clinician visits for identification of COPD exacerbations and for the correct assessment of clinical risk of COPD, as the app offers the possibility to regularly record relevant health data of a patient’s condition and symptoms. A strategy that could prove useful as several studies suggest that close to half of all exacerbations remain unreported. The unreported exacerbations and consequent lack of treatment by a health care professional were associated with worsening quality of life and increased risk of hospitalization.

### Potential Strengths and Limitations

A limitation that must be considered in the interpretation of the results is that it provides us information about how app users will perform within the context of a research study as the benefit perceived by the patient is a determining factor in the motivation to use the app. During the study, no decisions were made based on the information recorded in the app. Participants were informed that if they felt ill, they should contact their regular physicians for advice. Other limitations of this study are that we have not evaluated other factors that seem to influence daily compliance and affect both the health status and access to health care, such as socioeconomic status, impact on activities of daily living, and education level. However, in the analysis of factors associated with reporting, we must keep in mind that access to health care is likely to be an independent risk factor for underreporting in the general population. Another potential limitation is that the responses were dichotomous; there were substantial floor and ceiling effects resulting in failure to identify some of the exacerbations because once a symptom is present, no further change will be recorded.

Another limitation is that the study examines a relatively small prospective group of 116 patients monitored for only 6 months. However, as the population was enriched with patients with frequent exacerbators, 262 exacerbations were analyzed, which were equal to a rate of 2.25 per person every 6 months. This event rate can be explained because the relatively high proportion of patients included immediately after hospitalization might have contributed to a higher exacerbation rate and a possible seasonal effect. Missing data in the daily diary were also related to interest. The combination of both missing data and ceiling effects could have resulted in failure to identify some of the reported exacerbations in the daily diary. Furthermore, the analysis used ignored possible differences in symptom trajectory (early recovery from some symptoms) and these differences might be related to reporting.

### Conclusions

This study evaluates compliance in the daily use of an mHealth app among patients with COPD having a documented history of exacerbations who are motivated in their self-care for long-term monitoring without a mentoring process or regular phone calls. The findings of this cohort study confirm that daily use of the Prevexair app is feasible and acceptable for reporting daily symptoms and medicine use among people with COPD who are motivated in their self-care because of frequent decompensations of their disease. In addition, this study shows that monitoring through the Prevexair app has the potential for implementation of self-care plans and offers opportunities for interventions in the treatment of patients at risk of frequent exacerbations, identifying symptoms and providing a better diagnosis of their chronic condition. Further research must be carried out to evaluate this strategy for the management of COPD in clinical practice. In the near future, mHealth apps will be a natural complement to health telematics and personal health records. They should be a part of a complete solution to address changes in health care provision, and they are particularly suitable for chronic disease prevention and management.

## Abbreviations

AECOPD: acute exacerbations of COPD
CAT: Chronic Obstructive Pulmonary Disease Assessment Test
COPD: chronic obstructive pulmonary disease
FEV1: forced expiratory volume in one second
GSK: GlaxoSmithKline
mHealth: mobile health
mMRC: modified Medical Research Council
